# Editorial: Health conditions outside the brain and the risk of brain aging and dementia

**DOI:** 10.3389/frdem.2024.1372536

**Published:** 2024-02-21

**Authors:** Darren M. Lipnicki, Galit Weinstein

**Affiliations:** ^1^Centre for Healthy Brain Ageing (CHeBA), Discipline of Psychiatry and Mental Health, School of Clinical Medicine, Faculty of Medicine & Health, University of New South Wales, Sydney, NSW, Australia; ^2^School of Public Health, University of Haifa, Haifa, Israel

**Keywords:** dementia, brain aging, menopause, racial disparities, health conditions, risk factors, prevention

Numerous health conditions outside the brain are associated with increased risk of cognitive impairment and dementia. Indeed, the Lancet Commission on dementia prevention, intervention, and care includes hypertension, obesity, diabetes and hearing loss among 12 modifiable risk factors that account for around 40% of dementia globally (Livingston et al., 2020). However, many other health conditions outside the brain may contribute to neuropathology, cognitive impairment and dementia, including non-alcoholic fatty liver disease (Lu et al., 2023), kidney disease (Drew et al., 2019), and sarcopenia (Maniscalco et al., 2024). Further investigation is needed to determine whether these and other conditions are independent risk factors for brain aging and dementia, and when during the lifespan they exert their greatest effect. It is also important to have reliable and easily implemented measures of health conditions outside the brain associated with dementia risk, to facilitate early detection and intervention. The five papers in this Research Topic address these issues in various ways, expanding our understanding of known risk factors, providing evidence for more novel risk factors, and describing new approaches to measurement.

Ferguson et al. examined whether cardiovascular risk factors over the life course mediated the association between race and dementia risk. With data from 4 US-based prospective cohorts, they show that black individuals generally had higher dementia risk as well as higher levels of body mass index (BMI), fasting glucose, systolic blood pressure, and low-density lipoprotein cholesterol in late life. The association of race with dementia risk was mediated by all the examined cardiovascular risk factors, with BMI being the strongest mediator, explaining 39.1% of the association, followed by fasting glucose, accounting for 11% of the association. These risk factors also mediated the link between race and cognitive performance. Targeting these cardiovascular risk factors may thus help reduce disparities in dementia risk between black individuals and white individuals.

Racial disparities in dementia also motivated (Thompson et al.) to investigate associations between routine primary care measures and later cognitive impairment and dementia among First Nations residents of the Torres Strait region of Australia, who have particularly high rates of dementia (Russell et al., 2021). While urine albumin/creatinine ratio is not a novel maker for dementia, it was confirmed in this study as positively associated with later cognitive impairment and dementia. This is important for this population, in whom a lack of services and cultural reasons are among reasons for dementia detection rates being low (Bradley et al., 2020). A routine measure like urine albumin/creatinine ratio being specifically associated with later dementia may facilitate early identification, and thus enable interventions, for at-risk individuals in this population.

The importance of practical measures associated with dementia are clear to De Anda-Duran et al., who consider them particularly useful in populations that are remote, poorly serviced, or otherwise under-represented as clinical patients or participants in research. To this end, they are developing a user-friendly digital platform operating on personal technologies that are now almost ubiquitous. The measures are wide-ranging, including cognition, sleep behavior, mood, motor activity, and balance, as well as cardiovascular factors. The platform has been tested in remote and urban areas of the US, and will be refined for application on a global scale.

The remaining two papers of the Research Topic address menopause, which for some women may promote neurological and cognitive decline, and convey an increased Alzheimer's Disease (AD) risk (Brinton et al., 2015). Than et al. used data from over 15,000 women in the UK Biobank to show that compared to premenopausal women, perimenopausal women had worse performance on three of five cognitive domains (i.e. reaction time, visual memory and attention/working memory) at baseline, and that postmenopausal women had worse performance on all five (i.e. additionally verbal-numeric reasoning and prospective memory). Longitudinal assessments revealed that the decline in cognitive performance was similar between menopausal statuses, except for a decline in reaction time which was slower among post- compared to premenopausal women. These findings are more comprehensive than earlier studies that typically found poorer performance only for limited domains cross-sectionally and suggest that there is a rapid decline in cognitive performance during the perimenopausal period. While changes in the estrogen are often considered to be underlie such findings, Nerattini et al. have demonstrated in humans for the first time that increases in gonadotropin levels during menopause may be implicated in the association between menopause status and cognition. They showed that serum gonadotropin levels, particularly high levels of follicle-stimulating hormone (FSH), were associated with increased amyloid-beta load and lower gray matter volume in frontal brain areas of midlife women at risk for AD (based on family history and/or APOE-4 genotype). The association between FSH and the imaging biomarkers was observed across the menopause transition, though more pronounced at the postmenopausal stage than at the perimenopausal stage.

In summary, the studies included in this Research Topic introduce new data-driven tools for brain health monitoring (De Anda-Duran et al.), and underscore the importance of various factors such as gonadotropin levels (Nerattini et al.) and menopause status (Than et al.) in cognitive health and neuropathology. The included research further highlights the contribution of cardiovascular factors, particularly BMI, to racial disparity in dementia (Ferguson et al.) and the potential implication of urine albumin/creatinine ratio as a risk factor for dementia in a remote, under-represented population (Thompson et al.). Taken together, these studies clarify how information on health conditions outside the brain and remote organ dysfunctions can be utilized to inform approaches to the identification of individuals at greater risk of dementia, and offer preventive strategies to reduce the burden of cognitive impairment and dementia in various populations.

## Author contributions

DL: Writing – original draft, Writing – review & editing. GW: Writing – original draft, Writing – review & editing.
